# A Regional Model Comparison between MODPATH and MT3D of Groundwater Travel Time Distributions

**DOI:** 10.1111/gwat.70024

**Published:** 2025-09-22

**Authors:** Emily A. Baker, Paul Juckem, Daniel Feinstein, David Hart

**Affiliations:** ^1^ Wisconsin Geological & Natural History Survey University of Wisconsin‐Madison 3817 Mineral Point Road Madison WI 53705; ^2^ United States Geological Survey Upper Midwest Water Science Center 1 Gifford Pinchot Drive Madison WI 53726; ^3^ Geosciences Department University of Wisconsin‐Milwaukee 2100 E. Kenwood Boulevard Milwaukee WI 53211

## Abstract

Groundwater quality changes in wells and streams lag behind changes to land use due to groundwater travel times. Two contaminant transport methods were compared to assess differences in their simulated travel time distributions (TTDs) to streams and wells in the Wisconsin Central Sands. MODPATH simulates advective groundwater flow with particle tracking, while MT3D simulates age‐mass using a finite difference solution without dispersion to allow for direct comparison of the two methods. MODPATH appropriately simulates groundwater TTDs from the water table to surface discharge but is subject to inaccuracies at weak‐sink well cells due to the flow‐model grid discretization and imprecise location of well discharge within well cells. MT3D better represents weak‐sink well cells since it removes mass in proportion to the prescribed pumping rate, although travel time within well cells is neglected. Conversely, MT3D's treatment of surface water boundary cells is not as accurate as MODPATH because mass should be removed from the water table rather than the full cell volume. MT3D simulations of TTDs can also be confounded by the instantaneous vertical distribution of mass introduced throughout recharge cells instead of at the water table, which initiates mass along deeper flow paths. We evaluated 9 MODPATH and 13 MT3D implementations, generating differences in median travel times of up to 18 years. Both methods have strengths and weaknesses, with MT3D better representing weak‐sink well cell behavior and MODPATH better representing surficial recharge and discharge. The effect of these characteristics on simulated TTDs, along with ideas for ameliorating method weaknesses, is discussed.

## Introduction

Estimating the travel time of groundwater and associated contaminants through an aquifer to discharge features is an important objective of many groundwater modeling studies. Groundwater travel time distributions (TTDs), which show the age distribution of groundwater discharging from the aquifer, are helpful for visualizing ranges in travel times since they are more informative than simple metrics, such as the mean age. Many groundwater modeling studies have used either Lagrangian or Eulerian methods to simulate groundwater contaminant transport. Lagrangian methods of fluid flow track the trajectories and velocities of individual particles, while Eulerian methods track the change in fluid properties (e.g., solute concentration) across a grid over space and time (Zheng and Bennett 2002). MODPATH (Pollock 1988; Pollock 2016) and MT3D‐USGS (Zheng and Wang 1999; Bedekar et al. 2016) are two well‐known codes that use MODFLOW (McDonald and Harbaugh 1988; Harbaugh 2005) outputs to simulate transport through an aquifer. MODFLOW is a groundwater flow model that uses finite difference (FD) methods to simulate the cell‐by‐cell flows and potentiometric heads of water in an aquifer (McDonald and Harbaugh 1988; Harbaugh 2005). MODPATH uses a Lagrangian approach to track particles through the model domain using the velocity vector field determined by MODFLOW, producing path lines and travel times associated with each individual particle. MODPATH only considers advective transport and does not account for dispersion along the flow paths (Pollock 1988; Pollock 1989; Pollock 2016). MT3D‐USGS is a solute transport code that solves the advection‐dispersion equation for contaminant mass movement within the aquifer using an Eulerian approach when the FD solver is selected (MT3D‐USGS will be referred to as MT3D through the rest of the paper).

Partially due to the difference in the solution methods (Lagrangian vs. Eulerian), boundary conditions are handled differently in MODPATH and MT3D. For example, MODPATH allows particles to be initiated at a specific location within a cell, while MT3D instantaneously distributes the solute across the whole cell. This means that if a contaminant is introduced through the recharge boundary, it starts at a defined location within a cell for MODPATH (typically the water table elevation), but is distributed throughout the cell if MT3D is used, making the choice of vertical cell size influential on contaminant distribution. To better understand how these differences between MODPATH and MT3D affect simulated travel times, both methods were applied to a regional scale aquifer system. This study expands upon the work of Gusyev et al. (2014), who compared simulated tritium concentrations and age distributions for streams in New Zealand using both methods. Their work found that both methods simulated similar tritium concentrations, but that the age distributions generated by MT3D were truncated (less young and less old water) compared with MODPATH. Similarly, this study assesses how differences between MODPATH and MT3D affect simulated age distributions. However, this paper highlights additional considerations and nuances, such as the handling of weak‐sink cells and mass loading to cells, for evaluating the methods' strengths and weaknesses for generating groundwater TTDs.

The regional scale model of the Wisconsin Central Sands region (Fienen et al. 2021), which uses MODFLOW‐NWT (Niswonger et al. 2011), was updated and repurposed (Juckem and Starn 2021) for this study. The updated model was used to assess the travel time of recharge through the aquifer system to improve our understanding of travel times of agriculturally derived nitrate in a region that is currently experiencing nitrate concentrations in groundwater above the maximum contaminant limit (MCL) of 10 mg/L‐N due to decades of extensive agricultural management (Romano et al. 2023; WDNR 2023).

## Background

The Central Sands region of Wisconsin is an important agricultural area, where over 30% of the land is used for agricultural purposes (Romano et al. 2023). Unfortunately, the shallow aquifer in the region is highly susceptible to contamination from nitrate and other pollutants released at the surface due to the high permeability of the glacial deposits (Romano et al. 2023). Due to the rural nature of the region, people rely on private wells for drinking water. The combination of intense agriculture, high aquifer permeability, and reliance on private wells has led to issues with the quality of the drinking water in the region. In the Central Sands, the concentration of nitrate in groundwater commonly exceeds the drinking water standard for nitrate as nitrogen of 10 mg‐N/L (Campbell et al. 2023; Romano et al. 2023; WDNR 2023). Furthermore, it was found that the probability of exceeding this standard in the region ranged from 50 to 70% in Public Land Survey System (PLSS) sections (1 mile^2^ area) where over 80% of the land was used for agriculture (Romano et al. 2023). Many homeowners and communities are now dealing with high nitrate concentrations in their drinking wells and trying to determine how long it would take for ongoing changes in agricultural practices to result in improvements in groundwater quality.

Because groundwater travel times are an important component of simulating the transport of solutes through groundwater, understanding their relationship is important for resource managers (Eberts et al. 2013). Particle tracking, through the use of tools such as MODPATH (Pollock 2016), is a common method for estimating groundwater travel times with numerical models. Convolution‐based particle tracking (Robinson et al. 2010) allows for the application of TTDs, in conjunction with a source loading history, to perform contaminant transport simulations without the use of a conventional contaminant transport model that solves the advection‐dispersion equation, such as MT3D (Zheng and Wang 1999; Bedekar et al. 2016). Conversely, Goode (1996) demonstrated the use of contaminant transport codes (MT3D) for simulating groundwater travel times through the concept of age‐mass. To date, it appears that only Gusyev et al. (2014) have performed a direct comparison of these two methods. This work provides further exploration of the effect of important settings and nuances on the solution for each method.

## Methodology

### Groundwater Flow Model

A groundwater flow model of the Central Sands region developed by the U.S. Geological Survey (USGS; Fienen et al. 2022) was modified for the current study as described below; detailed information on the study area can be found in Section 1 of the Supporting Information. The regional model was developed using the Newton–Raphson formulation of the USGS modular finite‐difference groundwater flow modeling package (MODFLOW‐NWT; Niswonger et al. 2011; Hunt and Feinstein 2012; Niswonger et al. 2022). The original MODFLOW‐NWT input files from which the current study was developed are available in the corresponding USGS data release (Fienen et al. 2021). Lateral flow boundaries (the rivers that surround the modeled region) are represented using the MODFLOW general head boundary package (GHB). Streams and drainage ditches in the model domain are represented using the MODFLOW streamflow routing (SFR) package. About 3400 high‐capacity wells (https://dnr.wisconsin.gov/topic/Wells/HighCap) are represented using the MODFLOW well (WEL) package. Private wells are not included in the model. The horizontal resolution of the model cells is 200 m, resulting in a uniform model grid of 572 rows and 533 columns. The elevation of the top of the model, representing the land surface, was sampled from a 10‐m digital elevation model (DEM; WDNR 2019). Four layers were used to represent the major hydro‐stratigraphic units in the region and vary spatially in thickness: three layers represent glacial sediments, and one layer represents sandstone bedrock. The USGS soil water balance (SWB) code (Westenbroek et al. 2018) was used to determine the net infiltration due to precipitation and irrigation. History matching was performed for the original model using the parameter estimation package for high‐performance computing software, PEST++ (White et al. 2021), using groundwater head and streamflow data throughout the region. Further details on the development and calibration of the original published model are documented in Fienen et al. (2022).

**Figure 1:**
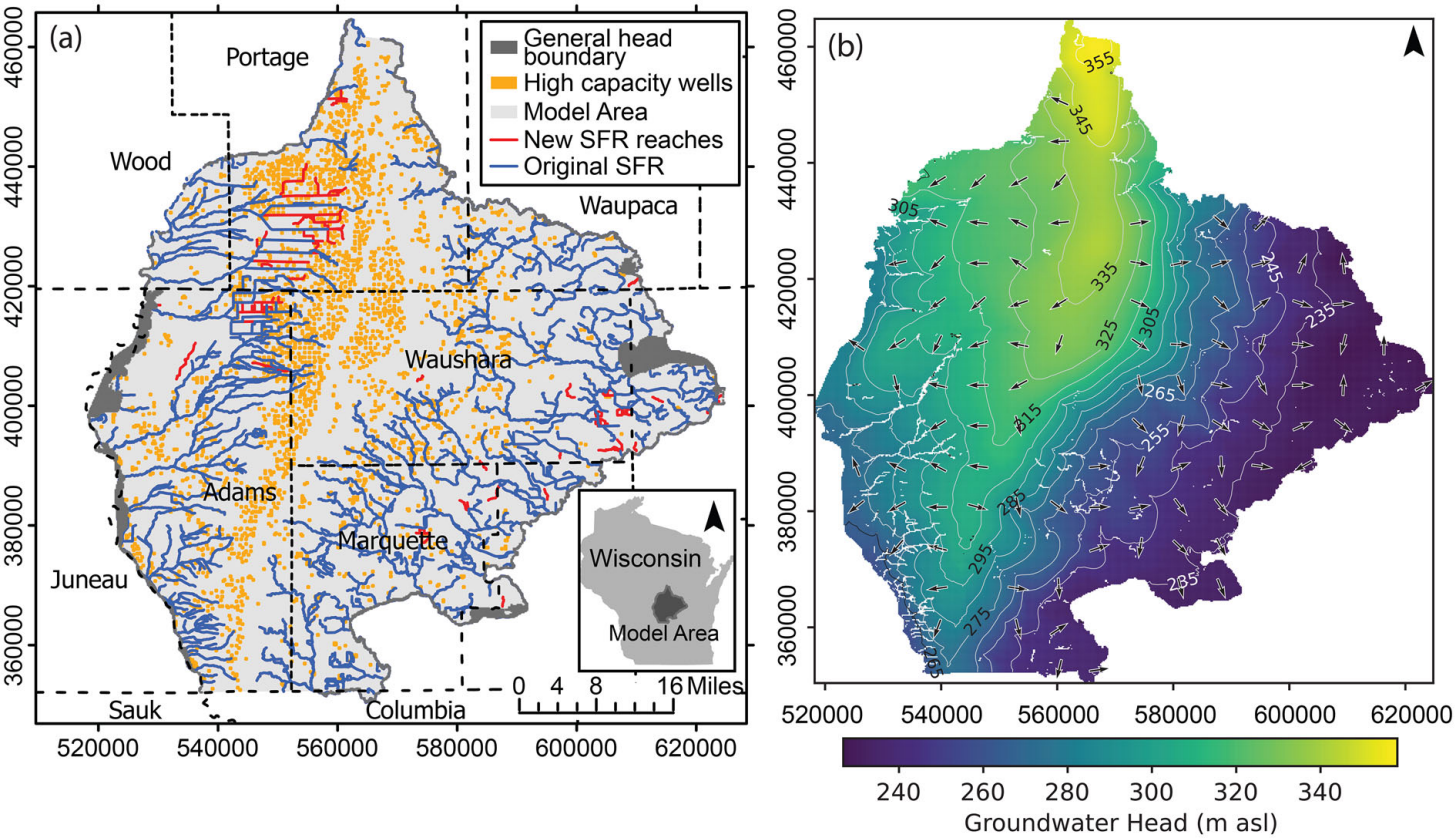
(a) Map of the Central Sands study area and the MODFLOW boundary conditions applied to the model. Streams and drainage ditches are represented using the MODFLOW streamflow routing (SFR) package. (b) Modeled groundwater heads with arrows depicting flow direction.

For this study, the SFR package representing the stream network was updated to incorporate smaller features, mostly drainage ditches, that are not included in the National Hydrography Dataset used to develop the original stream network. The stream network was updated using a 10 m resolution DEM (WDNR 2019), the National Hydrography Dataset version 2 (NHDPlusV2, VPU 04 and VPU 07) and field knowledge of the study area. Manual edits to the hydrography were made in ArcGIS Pro. The new SFR package input file was created using SFRmaker (Leaf et al. 2021a; Leaf et al. 2021b) run through FloPy (Bakker et al. 2016; Bakker et al. 2022). The newly added SFR reaches were assigned a streambed conductivity value of 1.0 m/d, equal to the median value of the original modeled reaches. The added SFR reaches are shown in Figure 1a and are located mainly in the northwest area of the model domain. In addition, to facilitate proper implementation of the IFACE setting in MODPATH (as detailed in the MODPATH Simulations section), cells located above SFR cells in layers two through four were set as inactive to ensure that the top of the SFR cells was adjacent to the model perimeter (Pollock 2016, page 19). The original published model consisted of a steady‐state stress period developed from mean 2012 through 2018 conditions, followed by monthly transient stress periods over this same time span (Fienen et al. 2022). The current study only uses the steady‐state period of the model to represent long‐term average flow conditions and simplify the analysis. This modified version of the Central Sands regional groundwater flow model by Fienen et al. (2022) is available from a HydroShare site (see the Data Availability Statement).

### MODPATH Simulations

An updated version of MODPATH version 7 (Pollock 2016) as implemented by Pérez‐Illanes and Fernàndez‐Garcia (2024) was used (without random walk) to assess the TTD from the water table to discharge locations (streams/rivers, drainage ditches, high‐capacity wells). MODPATH operates by using the flow vector field calculated in MODFLOW to produce particle path lines with associated travel times; we only considered advective transport. Multiple MODPATH simulations were run to assess how differences in the MODPATH setup affected calculated TTDs. Particles were placed in the top model layer in all cells that did not contain general head boundaries (since the GHB cells were only used to represent the large rivers at the edges of the model domain). Additionally, for the MODPATH simulations, the standard drape setting was used so that if the top cell was dry, the starting particle location was moved to the next wet cell below. Particles were started at the top of the saturated zone, that is, at the simulated water‐table elevation, unless otherwise noted, and were forward tracked until terminated by discharging out of the model domain. Test simulations were run by varying the number of particles placed in each water table cell (1, 5, 25, and recharge flux weighted), varying the vertical placement of the particles in the water table cells (top, center, bottom, or distributed throughout the cell), and changing the behavior of particles when they arrive at modeled weak‐sink cells (stop or pass through). For simulations with 1 particle per cell, the particle was placed in the horizontal cell center; for 5 particles per cell, a particle was placed in the horizontal cell center and the center of each quadrant; and for simulations with more than 5 particles per cell, the horizontal placement of the particles was random. When particles were flux weighted, the cells with the lowest recharge rate were assigned a single particle. It is important to note that in unconfined water table cells, MODPATH only considers the saturated thickness. Thus, starting particles at the “top” equates to starting particles at the water table (starting at the center means halfway between the water table and cell bottom).

Simulated sinks occur at boundary condition cells where groundwater discharges from the aquifer (e.g., SFR and WEL cells). *Strong‐sink cells* occur where groundwater enters the cell along all faces, whereas *weak‐sink cells* allow some water to pass out of the cell to another aquifer cell along at least one cell face (Zheng 1994; Abrams et al. 2012). The presence of weak‐sink cells can cause complications with MODPATH since it can be unclear whether specific particles should be discharged from the aquifer at the sink cell or pass through the cell and remain in the aquifer (Pollock 1994; Zheng 1994). The smaller the model grid discretization, the less likely it is that weak‐sink well cell problems occur, but even in a fairly refined model such as the Central Sands model (200 m grid spacing), weak‐sink cells are likely.

To mitigate this weak‐sink cell issue, the IFACE parameter was set to 6 in the MODFLOW input file for the SFR and GHB packages, and the MODFLOW cell‐by‐cell output was saved to a compact budget file. This design ensured that particles exiting through the upper cell face of a surface water boundary cell were captured, as per Abrams et al. (2012). For this setting to work properly, all non‐boundary package cells that overlaid boundary package cells (where SFR or GHB cells were placed in layers 2–4) were set as inactive cells in the IBOUND array of the MODFLOW BAS input file.

The WeakSinkOption (Pollock 2017) was set to 2 or “stop at” for most simulations, but changed to 1 or “pass through” for select simulations (Table 1). Thus, all particles entering a WEL package cell were either stopped or passed through, depending on the WeakSinkOption setting. The WeakSinkOption only affected the stopping of particles at weak‐sink well cells because IFACE was specified for all other boundary packages, which overrides this setting. For all MODPATH simulations, the porosity was set to a uniform value of 0.2 throughout the whole aquifer system in order to focus on the difference in the simulated MODPATH and MT3D TTDs.

**Table 1:** MODPATH Configurations and Transit Times for the Particle Tracking Simulations

						Percent of Particles Discharged at Time					
Run ID	MODPATH Settings	Particle Cell *z* Location	Number of Particles per Cell	Behavior at Weak Sinks	Time (Years) When 50% of Particles Are Discharged	1 Year	5 Years	10 Years	20 Years	50 Years	100 Years
MP‐1	Pass through weak sinks	Top (*z* = 1.0)	5	Pass through	23.9	5.0	20.4	32.2	46.3	65.2	78.3
MP‐2	Pass through weak sinks—flux weighted	Top (*z* = 1.0)	Varies	Pass through	25.0	5.1	19.9	31.4	45.4	64.2	77.3
Stop at weak sinks:											
MP‐3	1 particle cell top	Top (*z* = 1.0)	1	Stop at	18.0	5.3	22.9	36.6	52.6	72.2	83.9
MP‐4	5 particles cell top	Top (*z* = 1.0)	5	Stop at	17.9	5.7	23.1	36.6	52.5	72.2	84.0
MP‐5	25 particles cell top	Top (*z* = 1.0)	25	Stop at	17.9	6.1	23.2	36.6	52.5	72.2	84.0
MP‐6	Flux weighted particles	Top (*z* = 1.0)	Varies	Stop at	16.9	6.2	23.7	37.7	54.0	73.6	85.1
MP‐7	5 particles cell center	Center (*z* = 0.5)	5	Stop at	17.6	8.0	25.5	38.2	52.8	71.6	83.6
MP‐8	5 particles cell bottom	Bottom (*z* = 0)	5	Stop at	24.6	5.5	20.5	31.4	45.4	66.2	80.4
MP‐9	Distributed particles	*z* = 0, 0.25, 0.5, 0.75, 1.0	25	Stop at	19.0	7.2	24.0	36.4	51.1	70.5	83.0

After running the MODPATH simulations, particles with travel times of zero were removed prior to generating the cumulative distribution function (CDF) of travel times, since these particles never entered the groundwater system due to initial placement in stream cells (Abrams 2013) or well cells that are present in layer 1. A check was also performed to ensure that the surface area occupied by sink cells was not too large relative to the streams they represent, which can lead to inaccuracies in the CDFs of the particle travel times (Abrams 2013). This was done by computing the ratio between the particles released on active sink cells to the total number of particles released into the model. If this ratio is low enough, the effect on the resulting CDFs is negligible. According to Abrams (2013), the CDFs of the travel times are only slightly affected if the ratio is <0.09 and indistinguishably affected if <0.05. For our analyses, the ratio ranged from 0.003 to 0.072, which means the effect of the surface area of the sink cells that represent streams is negligible and therefore the travel times were not scaled (Abrams 2013). In addition to generating the CDFs of the travel times of the particles, the median travel time was calculated for each simulation along with the percent of particles discharged from the model domain after 1, 5, 10, 20, 50, and 100 years to assess transport through the aquifer at both a short timescale and over the timescale of historical nitrate use.

### MT3D Simulations

MT3D (Zheng and Wang 1999; Bedekar et al. 2016) simulations were also run to compare results to those from the MODPATH simulations by simulating the age‐mass distribution of groundwater TTDs. The concept of using mass to track water age was introduced by Goode (1996) and assumes mass is conserved, allowing for non‐solute specific simulation of groundwater age. For our model, a known mass is added, and then the time required for this mass to be discharged from the model is determined. This was done by loading mass into water table cells during an initial loading step and then simulating in subsequent steps the transport of that mass through the aquifer to its discharge location at surface water bodies and wells. A concentration of 1.0 (generic units) was assigned to the recharge that entered the aquifer during the loading step. This results in initial mass at all locations except where there are GHB cells since there is no recharge applied to GHB cells. Concentrations after 1 d of adding mass into water table cells were typically used as the starting concentration distribution for the MT3D simulations. The time step used to generate the starting concentration distribution was varied to assess whether this affected the resulting age‐mass TTDs. The age‐mass TTDs were calculated using the MT3D *.mas file, which provides the total solute mass in the model through time. First, the percent of the original solute mass remaining in the model through time was calculated from the *.mas file, and then this was converted to the percent of the original mass that has been discharged from the model over time to obtain the CDF of the age‐mass TTD of the model domain. A uniform porosity value of 0.2 was again assigned to all model cells in the aquifer system for comparison to MODPATH results. The DRYCELL option was set to True in the Basic Transport package (BTN) file to ensure mass is conserved through dry cells since MODFLOW‐NWT was used to simulate flow (Bedekar et al. 2016). The longitudinal dispersivity (α_L_) in the dispersion package was also varied to determine the magnitude of its effect on the MT3D age‐mass distribution. Lastly, MT3D was run using the stream flow transport (SFT) package to assess whether the transport of mass through the SFR boundaries affected the resulting age‐mass distribution.

In total, 9 MODPATH and 13 MT3D runs were assessed in this study. The modified MODFLOW input files and Jupyter Notebooks with example FloPy scripts for both the MODPATH and MT3D simulations are available on HydroShare (Baker et al. 2025) and provide additional information on the model setup.

## Results and Discussion

The updated regional Central Sands groundwater model produced comparable simulated heads and fluxes to the original model. Additional details are documented in Section 2 of the Supporting Information.

### MODPATH Particle Travel Times

**Figure 2:**
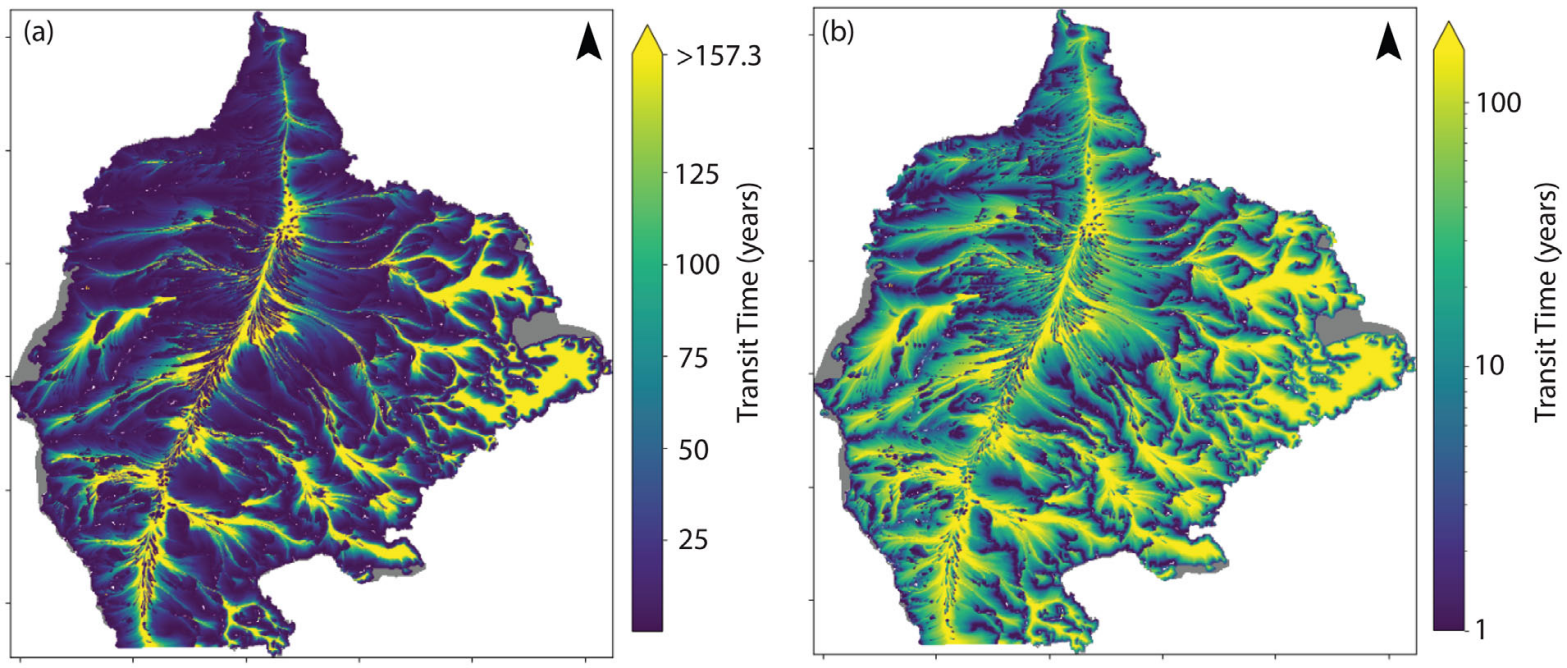
Spatial distribution of MODPATH particle transit times. Starting locations are shown, with color indicating transit time. Five particles were placed at the water table in each cell to simulate aquifer recharge. The color bar maximum is the 90th percentile of transit time (157.3 years). (a) Travel times using linear color bar. (b) Travel times using logarithmic color bar to highlight longer transit times.

Particles that recharge the groundwater system near the divide (center of the model domain) generally have relatively long travel times (90+ years), unless they are intercepted by one of the high‐capacity irrigation wells (Figure 2). Particles in the eastern area of the model domain can also have long travel times (Figure 2) due to the presence of glacial lake clay sediments and lower hydraulic gradients in this area. Most particles (~72%) are discharged through the SFR boundaries, which represent streams and irrigation ditches, while fewer particles (<7%) are discharged into the major rivers that border the model domain (GHB cells). Half of the particles released at the water table elevation are discharged from the aquifer within 16.9 years, and 85.1% of particles are discharged within 100 years (Table 1; MP‐6). Figure 2 shows the spatial distribution of all particle travel times based on MP‐4 according to their starting position in the model domain.

**Figure 3:**
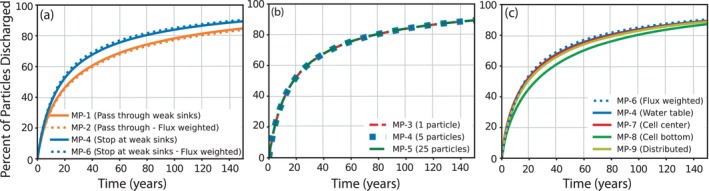
MODPATH particle transit time distributions through the Central Sands aquifer system varying (a) the particle behavior at weak sinks (pass through versus stop at weak sinks) and the recharge flux‐weighting scheme for particle placement, (b) the number of particles released per cell, (c) the starting vertical location of the particles. Particles are released at the elevation of the water table for all simulations plotted in (a) and (b). Refer to Table 1 for additional details on simulations MP‐1 to MP‐9.

Additional MODPATH simulations were run to assess how various modeling options influence the particle TTDs (Figure 3, Table 1). Such factors included the number and distribution of starting particles within model cells, and the treatment of particle discharge to weak‐sink cells. The results of these simulations show that if particles are allowed to “pass through” weak‐sink well cells, the particle TTD includes systematically older water (Figure 3a) with 25 years needed for 50% of particles to be discharged from the model (Table 1: MP‐2) compared with 17 years when particles are “stopped at” weak‐sink well cells (Table 1: MP‐6). As discussed previously and discussed by Abrams et al. (2012), the proper setting for the WeakSinkOption for weak‐sink well cells is unclear; the “correct” simulated age distribution from a sufficiently refined model in which all well cells are strong sink cells would plot somewhere between the MP‐2 and MP‐6 lines in Figure 3a. When the number of particles started in each model grid cell at the water table is varied from 1 to 25 particles per cell, the resulting age distributions are nearly identical (Table 1: MP‐3, MP‐4, MP‐5; Figure 3b), indicating that particle density is inconsequential for this regional analysis.

Lastly, particles tend to discharge from the model sooner when the particles are initially placed at the elevation of the water table (Figure 3c and Table 1: MP‐4) compared with particles initially placed closer to the bottom of the grid cell (Table 1: MP‐8, MP‐9). In recharge‐controlled settings (Haitjema and Mitchell‐Bruker 2005) where flow is primarily horizontal, such as the Central Sands of Wisconsin, this pattern is likely because particles that are initiated deeper within water table cells travel along deeper flow paths and travel a greater horizontal distance through the aquifer before discharging to surface water boundaries that tend to be further away from their starting location compared with particles that start at shallow depths and follow shallow flow paths prior to discharging at nearby surface boundaries (Abrams et al. 2012). When the particle starting locations are distributed vertically throughout the entire grid cell (top, 0.25, 0.5 and 0.75 of the saturated cell thickness, and bottom), the TTD is similar to that produced when particles are initially placed at the vertical center of the saturated thickness (Table 1: MP‐9; Figure 3c). This is expected given the longer travel times of the particles placed at the cell bottom and shorter travel times of the particles placed at the water table. Starting particles at depths below the water table is unrealistic behavior given that recharge enters the aquifer at the water table; however, doing so provides a useful comparison to subsequent MT3D simulations.

### MT3D Age‐Mass Distributions

**Figure 4:**
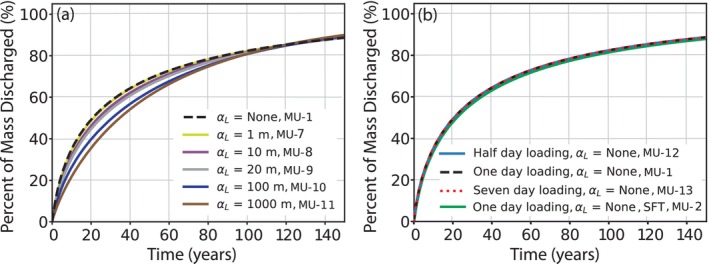
MT3D‐USGS age‐mass distributions for 150‐year simulation period. (a) Age‐mass distributions calculated with different values of longitudinal dispersivity (α_L_) using the finite difference (FD) solver. (b) Age‐mass distributions implementing the stream flow transport (SFT) package and varying the duration of the initial mass loading period without dispersivity using the FD solver. Refer to Table 2 for additional details on simulations MU‐1 to MU‐13.

The modified Central Sands regional groundwater model was also used to perform MT3D simulations to calculate the age‐mass TTDs for groundwater recharge traveling through the aquifer to discharge locations. Initial simulations showed that under certain MT3D configurations, negative concentrations were calculated in certain model cells due to cross dispersion. To minimize this issue, the “NOCROSS” keyword in the MT3D dispersion package was implemented, which sets the cross‐dispersion terms to zero, which is appropriate for highly advection dominated simulations (Bedekar et al. 2016). The base case MT3D simulation uses the finite‐difference solver (with MIXELM = 0) and only simulates advection (no dispersion) to directly compare the results to the MODPATH simulations. This simulation estimates that 50% of the starting mass is discharged from the aquifer in 20.7 years and 82.4% of the mass is discharged in 100 years (Table 2: MU‐1). The dispersion package was then added to assess the effect of dispersion, with longitudinal dispersivity values (α_L_) from 0.0001 to 1000 m tested (Table 2: MU‐3 through MU‐11). For small values of α_L_ (<1 m), the results are similar to MU‐1 in which dispersion was ignored and are therefore not shown in Figure 4a. For higher α_L_ values, the percent of the starting mass discharged from the system was smaller for the beginning and middle periods of the travel time range, became similar to the base case simulation of no dispersion around 120 simulated years, and was slightly greater for travel times longer than 120 years (Figure 4a). That is, the effect of hydrodynamic mixing appears to be most pronounced around median travel times (up to 14 years difference among MU‐11 with α_L_ = 1000 m compared with MU‐3 with α_L_ = 0.0001 m, which are extreme values used primarily for illustration; Figure 4 and Table 2). For the tested α_L_ values of 100 and 1000 m, the grid Peclet number is ≤2, at which point dispersion dominates and numerical dispersion is secondary for transport simulations (Zheng and Wang 1999). For tested α_L_ values of 0.0001 m through 20 m, the grid Péclet number is larger, giving rise to the possibility that numerical dispersion distorts some results. While numerical dispersion can be particularly problematic for simulating sharp concentration fronts, it is of less concern for this study since the focus is on primarily advective transport of a regionally extensive contaminant that is broadly sourced. Moreover, numerical dispersion is shown to have minimal influence in this study because α_L_ values less than 1 m (MU‐3 through MU‐7) are indistinguishable from the simulation with no dispersion (MU‐1) at the scale of analysis (Figure 4a.).

**Table 2:** MT3D‐USGS Configurations and Age‐Mass Results for Solute Transport Simulations Using the Finite‐Difference (FD) Solver

MT3D‐USGS Settings				Percent of Mass Discharged at Time					
Run ID	Solver	α_L_ (m)	Time (Years) When 50% of Mass Is Discharged	1 Year	5 Years	10 Years	20 Years	50 Years	100 Years
MU‐1	FD	None	20.7	6.6	23.1	35.0	49.3	69.1	82.4
MU‐2	FD, SFT	None	21.9	6.3	22.2	33.9	48.0	68.0	81.5
MU‐3	FD, NOCROSS	0.0001	20.7	6.6	23.1	35.0	49.3	69.1	82.4
MU‐4	FD, NOCROSS	0.001	20.7	6.6	23.1	35.0	49.2	69.1	82.4
MU‐5	FD, NOCROSS	0.01	20.7	6.6	23.1	35.0	49.3	69.1	82.4
MU‐6	FD, NOCROSS	0.1	20.8	6.6	23.1	34.9	49.2	69.1	82.4
MU‐7	FD, NOCROSS	1	21.2	6.5	22.5	34.3	48.7	68.9	82.3
MU‐8	FD, NOCROSS	10	23.6	5.9	20.5	31.9	46.3	67.2	81.8
MU‐9	FD, NOCROSS	20	25.2	5.6	19.5	30.5	44.7	66.0	81.4
MU‐10	FD, NOCROSS	100	30.8	4.7	16.6	26.4	39.8	62.8	80.8
MU‐11	FD, NOCROSS	1000	34.6	3.5	12.8	21.8	35.6	60.9	80.9
MU‐12	FD, 1/2 day load	None	20.7	6.6	23.2	35.0	49.3	69.1	82.4
MU‐13	FD, 1 week load	None	20.7	6.6	23.1	34.9	49.2	69.1	82.4

Note: NOCROSS indicates that the NOCROSS keyword is used in the dispersion package to eliminate cross‐dispersion terms that produce negative concentrations.

Lastly, the potential for streams to capture and then re‐infiltrate age‐mass downstream (via the SFT package; MU‐2) as well as sensitivity to the duration of the initial mass loading step was assessed by varying the loading duration from half a day to 1 d to 7 d (Table 2: MU‐12, MU‐1, MU‐13, respectively). These modifications were found to have minimal effect on the age‐mass distribution over the subsequent 150‐year time period, with all three TTDs essentially overlapping (Figure 4b). All three loading scenarios that explored the initial time for adding mass had the same time required for 50% of particles to be discharged from the model (20.7 years) and their percentages of mass discharged at each assessed time were either the same or within 0.1 years, revealing limited sensitivity to this factor over the time steps explored (Table 2; MU‐1, MU‐12, MU‐13). Meanwhile, the use of the SFT package led to differences in the timing of particle discharge of about 1 year. The time for 50% of mass to be discharged and the percent of mass discharged through the model boundaries at six time intervals from 1 to 100 years for all of the MT3D configurations are provided in Table 2.

### MODPATH – MT3D Comparison

**Figure 5:**
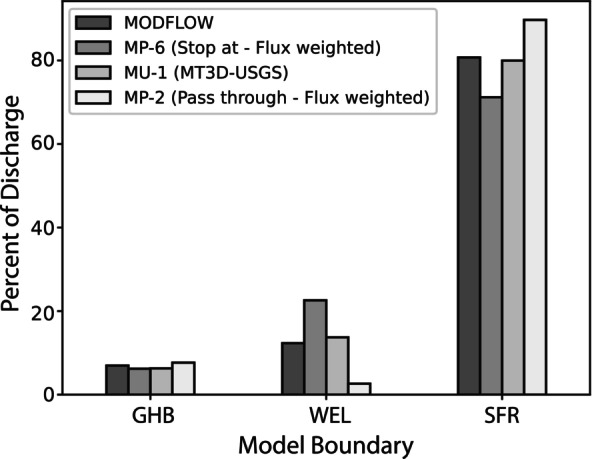
Comparison of the percent of groundwater discharged through each boundary condition for the steady‐state flow model to the percent of age‐mass (MT3D‐USGS; MU‐1) and percent of particles (MODPATH) discharged through each boundary at 50 years. MODPATH simulations for both treatments of weak sink cells (pass‐through weak sinks = MP‐2; stop‐at weak sinks = MP‐6) are shown. GHB is the general head boundary package, SFR is the streamflow routing package, and WEL is the well package of MODFLOW.

MODPATH and MT3D results were directly compared in two ways: first, by the percent of age‐mass and particles removed by each boundary package (sinks), and second, by comparing TTDs. Groundwater discharge through each MODFLOW boundary type (SFR, GHB, WEL) was compared to the flux of mass and particles through each of those boundary types (Figure 5). MODPATH simulations started particles at the water table, with the particle density weighted by recharge flux per cell. However, both the “pass through” (MP‐2) and “stop at” (MP‐6) settings for WeakSinkOption were used for the comparison. The amount of water (MODFLOW), amount of age‐mass (MT3D), and number of particles (MODPATH) discharged through each boundary were divided by the total water, mass, and particles, respectively, removed by all three boundaries at the 50‐year time interval to obtain the percentage of water, mass, and particles discharged through each boundary (Figure 5). The greatest discharge of water (80.7%; MODFLOW) occurred through the SFR boundary cells, which represent the streams and drainage ditches throughout the interior of the model domain. The remaining water discharged from the MODFLOW model was through GHB cells (7.0%), which represent the major rivers at the model edges, and through well cells (12.3%). The percent of the MT3D age‐mass that discharged through each of the boundary conditions was similar to the water discharged through each boundary (SFR 80.0%, GHB 6.3%, and WEL 13.7%). The percent of MODPATH particles discharged through each boundary type showed a greater discrepancy with the groundwater flux through each boundary type and varied depending on the behavior of particles at weak‐sink well cells. The MODPATH simulation with WeakSinkOption set to “stop at” (MP‐6) overestimated the number of particles discharged through wells at the expense of particles discharged through streams; the opposite occurred for the simulation with the WeakSinkOption set to “pass through” (MP‐2, Figure 5). This difference between the MODPATH simulations and those compared with MODFLOW and MT3D is due to the abundance of weak‐sink well cells simulated by the model and the binary nature of the WeakSinkOption. That is, the WeakSinkOption in MODPATH is uniformly applied to all weak‐sink well cells, so all particles that pass through such cells are either passed through or stopped and captured, even though not all groundwater in a weak‐sink well cell is removed by MODFLOW. This not only affects where particles are terminated, but the age of the particles as well, in that some “passed through” particles will travel further than appropriate before discharging to down‐gradient streams, whereas some “stopped at” particles will stop prematurely at weak‐sink well cells. Similarly, while MT3D discharges age‐mass proportionally to the water discharged by MODFLOW, the age of the mass may not match with all conceptualizations of the flow system. That is, simulated age‐mass is discharged uniformly across each boundary cell volume (appropriate for wells) rather than across cell faces, as should be the case for boundary packages that represent surficial features such as the SFR package (and the GHB package in this Central Sands model). Thus, MT3D better represents the amount and age of mass discharged to weak‐sink well cells than MODPATH, but MODPATH better represents the age of particles discharged to surface boundary packages when IFACE is properly implemented.

**Figure 6:**
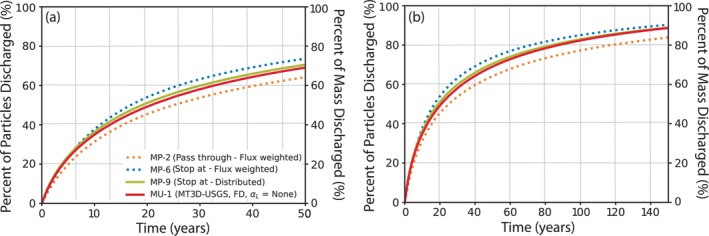
Comparison of select transit time distributions of recharge through the aquifer system determined using MODPATH and MT3D‐USGS for (a) 50 years and (b) 150 years.

Direct comparisons among MODPATH and MT3D simulated TTDs highlight the ways in which starting and stopping particles and age‐mass influence results. MODPATH particle TTDs for selected cases are graphically compared with the MT3D age‐mass TTD for the case with no dispersion (MU‐1) in Figure 6. The influence of how particles or mass are initiated, or started, in the simulations is best evaluated by comparing two MODPATH simulations that stop particles at weak‐sink well cells, but either initiate particles at the water table (MP‐6) or distributed throughout the water table cell (MP‐9) similar to the way that MT3D initiates age‐mass. The influence of how particles and mass are stopped at sinks is illustrated by comparing the two MODPATH simulations with different WeakSinkOption settings (MP‐3 and MP‐6), as well as by comparing the MODPATH simulation that initiated particles throughout water table cells (MP‐9) with the MT3D simulation (MU‐1). The influence of how particles and mass are initiated is discussed below first, followed by a discussion of how the means by which particles and mass are stopped influence TTDs; ideas for minimizing the associated challenges are also discussed.

As described previously in the “MODPATH Particle Travel Times” Section, starting particles (or age‐mass) below the water table, as simulated by run MP‐9, results in TTDs that are biased old compared to when particles are started at the water table (MP‐6). This bias is the result of initiating particles, or mass, along inappropriately deep flow lines that discharge to more distant sinks, most commonly, large river systems in the Central Sands model. This is well illustrated by Figure 2, in which particles that start near the groundwater divide (central north–south line highlighted by shades of yellow) where downward vertical hydraulic gradients are steepest tend to reach the greatest depths and thus travel the greatest distance over the longest time intervals before discharging. Like run MP‐9, MT3D also instantly distributes mass entering via recharge throughout the full thickness of the water table cells rather than placing the mass at the elevation of the water table surface. This problem of instantaneous mass distribution could be lessened by refining or introducing a thin surficial model layer that mirrors the contours of the simulated water table, thus spreading introduced mass over a smaller volume for MT3D simulations. This is essentially a manual approximation of how a recently updated Method of Characteristics (MOC) technique (Winston et al. 2018) automatically addresses this limitation in MT3D (this updated MOC method is not compatible with MODFLOW‐NWT). Refining a single layer may also be an appealing compromise over refining the entire model grid because finer resolution grids often result in increased run times (MT3D simulations took 12.5 h or longer on a Windows computer with a 2.90 GHz Intel® Gold 6226R CPU processor with 96 GB RAM while MODPATH simulations took about 4 min when 5 particles were placed in each grid cell [MP‐4; 742,555 tracked particles] and 4 h for the flux‐weighted particle simulations (MP‐6; 203,570,389 tracked particles) when using the standard forward‐tracking solution available the updated version of MODPATH version 7 (Pérez‐Illanes and Fernàndez‐Garcia 2024)).

The second reason for differing TTDs is due to differences in how MODPATH and MT3D capture (remove) particles or mass from model cells at discharge locations, or sinks. This phenomenon is most evident when comparing MODPATH simulations that either stop particles at weak‐sink well cells (MP‐6) or allow them to pass through (MP‐2). That is, MODPATH captures individual particles that intersect specified cell faces in a way that matches natural flow processes when IFACE is specified using values associated with the location of boundary packages (IFACE = 1 to 6). As Abrams et al. (2012) pointed out, stopping particles at the top cell face of weak‐sink cells is the appropriate setting for surface boundary conditions. However, for internal sinks (discharge that does not occur at a cell face), such as wells, use of the WeakSinkOption results in the capture of either all particles or none, depending on whether the WeakSinkOption is set to “stop at” or “pass through.” MT3D captures mass on a groundwater‐flux proportional basis throughout the entire cell volume. For example, if 50% of water in a cell is removed by a boundary package in MODFLOW, 50% of mass in MT3D is removed throughout the cell volume, regardless of the relative location of the mass within the cell. As illustrated in Figure 5, MT3D is therefore better able to match the age‐mass discharge at weak‐sink well cells to the amount of water discharged from these cells by MODFLOW than are MODPATH simulations, regardless of the WeakSinkOption setting. Conversely, MT3D's cell‐wide removal of age‐mass from weak‐sink stream cells is non‐ideal because age‐mass is not removed along the top face of stream cells. Nonetheless, similarities between the MT3D TTD (MU‐1) and the TTD of the initially distributed MODPATH run (MP‐9) may imply that at a regional scale, MT3D's method of capturing age‐mass may reasonably approximate the “stop at” setting for the WeakSinkOption in MODPATH. This is further noted by the fact that as ages increase, especially beyond about 60 years, the MT3D TTD approaches the MODPATH simulation with particles started at the water table and “stopped at” weak‐sink well cells (MP‐6).

Combined, these patterns highlight how the simulated introduction and removal of particles or mass influence simulated travel times for contaminants, such as nitrates, that primarily enter and leave aquifers across the water table. Understanding the strengths and weaknesses of each method can aid in selecting the more appropriate tool for a particular application or with identifying potential approaches for minimizing the respective weaknesses. As suggested earlier for minimizing the spreading of introduced age‐mass throughout water table cells, refining or introducing a thin layer that mirrors the water table and only extends a small thickness below surficial boundary cells (streams) could minimize both issues (not introducing or removing mass directly at the water table) associated with MT3D's cell‐average treatment of age‐mass. Similarly, refining all model layers would minimize age biases from weak‐sink well cells for MODPATH because, as cell size decreases, total flow through individual cells tends to decrease and well pumping becomes a greater portion of total flow, resulting in stronger weak‐sink cells. Indeed, the greater similarity between MT3D and MODPATH TTDs illustrated in this study compared with that of Gusyev et al. (2014) may be partially attributable to the representation of surficial unconsolidated sediments in the Central Sands model by three relatively thin layers. Alternatively, analytical methods for tracking particles within weak‐sink well cells (Starn et al. 2012; Rhamadhan 2015; Muffels et al. 2017) or the use of numerical inset models of weak‐sink well cells with sufficient grid resolution to ensure the well cell is a strong sink (Spitz 2001; Paschke et al. 2007) could be employed to mitigate weak‐sink well cell limitations with particle tracking. Another option for improving weak‐sink well cell handling by MODPATH would be to utilize zones whereby cells in which well pumping exceeds a percentage of total inflow to the cell would be identified as cells where all particles should be stopped, whereas well cells with pumping below this threshold would allow all particles to pass. This is similar to prior versions of MODPATH (versions 5 and earlier) that had a setting for stopping or passing particles at weak‐sink cells based on a weak‐sink “threshold,” or the ratio of discharge via the sink divided by the total inflow to the cell. All particles intersecting a cell with a sufficiently weak‐sink (less than the threshold) were passed through; all particles intersecting a cell with sink discharge greater than the threshold were stopped. While this functionality is not available in MODPATH versions 6 and later, the method could be replicated by preprocessing the budget file and generating stop zones. However, determining an appropriate fractional weak‐sink threshold remains subjective as it is not equivalent to removing particles in proportion with the amount of water discharged from the cell.

## Conclusions

In this study, the Central Sands Lake Study groundwater flow model (Fienen et al. 2022) was updated and used to analyze the TTD of groundwater from recharge at the water table to discharge along rivers and wells in the region to compare results between Lagrangian (MODPATH) and Eulerian (MT3D) methods. For the current model design, both MODPATH and MT3D have evident strengths and weaknesses. MODPATH is well suited for simulating groundwater age from the water table to surface discharge but is challenged by the behavior of particles at weak‐sink well cells. MT3D simulations of TTDs are challenged by the instantaneous vertical distribution of age‐mass throughout the recharge cell, such that cell dimensions can affect travel times, but age‐mass discharge into weak‐sink well cells is better represented because mass withdrawal is flux‐based, as is pumped water withdrawal in the underlying MODFLOW code. MT3D limitations could be reduced by introducing a thin surficial layer to simulate the water table, thereby refining the cell volume over which most of the simulated mass is introduced and removed from the aquifer. MODPATH limitations with weak‐sink well cells could be reduced by incorporating analytical (Starn et al. 2012; Rhamadhan 2015) or numerical (Spitz 2001; Paschke et al. 2007) methods for improved particle tracking in weak‐sink well cells, or by using zones to distinguish between relatively weak and strong weak‐sink well cells. Alternatively, modelers may consider using mod‐PATH3DU (Muffels et al. 2017), which employs a semi‐analytical method (Rhamadhan 2015) that automatically improves how particles passing through weak sink cells are handled. Additional directions for future work include simulating dispersion with MODPATH (Pérez‐Illanes and Fernàndez‐Garcia 2024), incorporating the unsaturated zone package (UZF) to simulate unsaturated zone travel times (Niswonger et al. 2006), and further study of denitrification rates occurring throughout the aquifer system so that nitrate transport through the aquifer can be more accurately estimated using the MT3D reaction (RCT) package or via convolution equations with age distributions from MODPATH (Maloszewski and Zuber 1982; McDonald et al. 2015; Juckem et al. 2024; Juckem and Green 2024). While both transport models have limitations, this study shows that both particle tracking (MODPATH) and solute transport (MT3D) have strengths and weaknesses, and can provide informative results on the TTD of water and associated regionally extensive contaminants through a regional aquifer system. Future studies should consider carefully the limitations discussed and choose the most appropriate method based on their model design and objectives.

## Authors' Note

The authors do not have any conflicts of interest or financial disclosures to report.

## Supporting information


**Data S1.** Supporting Information.

## Data Availability

Model files that support the findings of this study are openly available through HydroShare at http://www.hydroshare.org/resource/767cc0e1b6ac41b4a5893bebbc1d126d.
