# Supplementary material for: A Regional Model Comparison between MODPATH and MT3D of Groundwater Travel Time Distributions
Source: Ground Water. 2025 Sep 22;63(6):861–73. doi: 10.1111/gwat.70024 (PMC12652280; doi:10.1111/gwat.70024)
Supplement: Supplementary file 1 — Data S1. Supporting Information. [file GWAT-63-861-s001.pdf]

Supporting Information: A regional model comparison between MODPATH and MT3D of groundwater travel time distributions

Emily A. Baker[1,2], Paul Juckem[3], Daniel Feinstein[4] and David Hart[2]

[1] Geosciences Department, Hamilton College
[2] Wisconsin Geological & Natural History Survey, University of Wisconsin-Madison
[3] Upper Midwest Water Science Center, U.S. Geological Survey
[4] University of Wisconsin-Milwaukee

Section 1. Study Area

The modeled study area consists of about 75% of the Central Sands region, extending across nine counties in central Wisconsin (Figure 1a). The western edge of the model domain borders the Wisconsin and Plover Rivers, while the northern and eastern edges border the Tomorrow, Waupaca, Wolf, and Fox Rivers. Multiple glacial end moraines extend from north to south dividing the study area along its center and forming a topographic high that delineates the hydrologic divide (Fienen et al., 2022), with streams to the west flowing west to the Wisconsin and Plover Rivers and streams to the east flowing east to the Tomorrow, Waupaca, Wolf, and Fox Rivers. Groundwater flow in the region behaves similarly, as shown by the flow vectors calculated in MODFLOW (Figure 1b). Drainage ditches have been constructed throughout much of the region, increasing the drainage density to lower the water table. Groundwater flowing through this surficial aquifer discharges predominantly to the interior network of streams and drainage ditches. Quaternary-aged coarse sand and gravel glacial sediments form the surficial aquifer throughout the region and consist of a combination of glacial lake, end moraine till, ground till, outwash, stagnant ice, and stream sediments (Kraft et al., 2012; Fienen et al., 2022). Thickness of the surficial aquifer varies greatly; where bedrock topographic highs occur, the glacial surficial aquifer can be thin or absent, while it can be over 100 m thick in other areas (Fienen et al., 2022; Bradbury et al., 2017). The horizontal hydraulic conductivity of the surficial aquifer is often in the range of 10 to 80 m/day, but varies with depth and can range from <1 m/day to more than 150 m/day over the study area. For example, lower hydraulic conductivity values are common near the eastern edge of the modeled area where fine-grained sediments were deposited by glacial Lake Oshkosh (Weeks & Stangland, 1971; Kraft & Mechenich et al., 2010; Kraft et al., 2012; Fienen et al., 2022). Underlying the glacial sediments is Cambrian-aged sandstone bedrock across the southern portion of the study area, with Precambrian crystalline rock across the northern portion of the area (Fienen et al., 2022; Bradbury et al., 2017). The Cambrian sandstones are considered aquifers but have a hydraulic conductivity an order of magnitude less than the overlying sediments (Fienen et al., 2022); the Precambrian crystalline rock is considered impermeable for this work. Additional information on the geology of the study area can be found in Fienen et al. (2022), the Wisconsin Department of Natural Resources Central Sands Lakes study report (WDNR, 2021: app. A), Bradbury et al., 2017, and Kraft et al., 2012.

Section 2. MODFLOW Results

Groundwater heads in the Central Sands region are highest along the center of the model domain along the groundwater divide and decrease to the east and west as groundwater flows toward the major rivers that coincide with the edges of the model domain (Figure 1b). The simulated groundwater heads and fluxes for the repurposed Central Sands regional groundwater model are very similar to those calculated by the original model (Fienen et al., 2022), with 88% of heads within 1 m. The larger differences in the calculated heads for the two models are concentrated around the new SFR reaches, especially in the northwest corner of the model domain in Portage County and in the northeast corner of Adams County where the majority of the new SFR cells are located. In these locations, the calculated heads using the new SFR cells are lower than in the original model due to the addition of stream reaches. The lowering of modeled heads in this area of the model with the addition of the new SFR cells improves model fit since the heads there were too high (and sometimes above the elevation of the land surface) in the original model configuration (Fienen et al., 2021). While this discrepancy was not important for the original model because it was only used to inform perimeter boundary conditions for smaller scale inset models, improving the heads in this area of the model domain was important for our purposes. Meanwhile, the percent of the groundwater fluxes through each of the model boundary types remained nearly the same (<1% change in percent of flux). The greatest percent flux of groundwater discharging the model continued to be through the SFR boundaries, at 80.7% of the flux, while the GHB and WEL boundaries accounted for 7.0% and 12.3% respectively of the groundwater flux leaving the model domain. Given that the only change to the model was the addition of the SFR cells depicted in Figure 1 to account for minor discharge areas, and that the modeled heads were improved where SFR reaches were added, a model recalibration in PEST was not performed.

References

Bradbury, K.R., M.N. Fienen, M.L. Kniffin, J.J. Krause, S.M. Westenbroek, A.T. Leaf and P.M. Barlow. 2017. A Groundwater Flow Model for the Little Plover River Basin in Wisconsin's Central Sands. Wisconsin Geological and Natural History Survey Bulletin 111, 82 p., at https://water.usgs.gov/GIS/dsdl/gwmodels/WGNHS2017-LittlePlover/WGNHS2017_B111-report.pdf

Fienen, M.N., M.J. Haserodt, A.T. Leaf, and S.M. Westenbroek. 2022. Simulation of Regional Groundwater Flow and Groundwater/ Lake Interactions in the Central Sands, Wisconsin, U.S. Geological Survey Scientific Investigations Report 2022–5046, 111 p., https://doi.org/10.3133/sir20225046

Fienen, M.N., M.J. Haserodt, and A.T. Leaf. 2021. MODFLOW models used to simulate groundwater flow in the Wisconsin Central Sands Study Area, 2012-2018, U.S. Geological Survey data release, https://doi.org/10.5066/P9BVFSGJ

Kraft, G. J., K. Clancy, D.J. Mechenich, and J. Haucke. 2012. Irrigation Effects in the Northern Lake States: Wisconsin Central Sands Revisited. Ground Water, 50(2), 308–318. https://doi.org/10.1111/j.1745-6584.2011.00836.x

Kraft, G. J., and D.J. Mechenich. 2010. Groundwater Pumping Effects on Groundwater Levels, Lake Levels, and Streamflows in the Wisconsin Central Sands, A Report to the Wisconsin Department of Natural Resources in Completion of Project: NMI00000247. https://waushara.extension.wisc.edu/files/2010/12/gwpumpcentralsands2010.pdf

Weeks, E.P., and H.G. Stangland. 1971. Effects of irrigation on streamflow in the Central Sand Plain of Wisconsin: U.S. Geological Survey Open-File Report 70-362, 113 p, accessed August 16, 2022, at https://doi.org/10.3133/ofr70362

Wisconsin Department of Natural Resources (WDNR). 2021. Central Sands Lakes study report—Findings and recommendations: Wisconsin Department of Natural Resources, Report to the Wisconsin Legislature, May 27, 2021, accessed June 4, 2021, at https://dnr.wisconsin.gov/topic/Wells/HighCap/CSLStudy.html#reports
